# Mucus hypersecretion in chronic obstructive pulmonary disease: From molecular mechanisms to treatment

**DOI:** 10.2478/jtim-2023-0094

**Published:** 2023-12-20

**Authors:** Ruonan Yang, Xiaojie Wu, Abdelilah Soussi Gounni, Jungang Xie

**Affiliations:** Department of Respiratory and Critical Care Medicine, National Clinical Research Center of Respiratory Disease, Key Laboratory of Pulmonary Diseases of Health Ministry, Tongji Hospital, Tongji Medical College, Huazhong University of Science and Technology, Wuhan 430030, Hubei Province, China; Department of Respiratory and Critical Care Medicine, Wuhan NO. 1 Hospital, Wuhan Hospital of traditional Chinese and Western Medicine, Wuhan 430022, Hubei Province, China; Department of Immunology, Faculty of Medicine, University of Manitoba, Manitoba R3E 0W3, Canada

## Introduction

Chronic obstructive pulmonary disease (COPD) is among the leading causes of mortality and morbidity worldwide, which has caused tremendous burden on society.^[1,2]^ Mucus hypersecretion is recognized as one of the main pathophysiological changes and is associated with lung function decline, exacerbations, hospitalization and mortality in patients with COPD.^[3]^ Exposure to cigarette smoke or other irritants promote submucosal gland hypertrophy and goblet cell metaplasia to overproduce mucus through the activation of a variety of signaling pathways. In turn, mucus accumulated in airways worsens the symptoms of airway obstruction and hypoxemia.^[4]^ With the function of blocking specific signaling pathway, it has been identified that drugs targeting mucus hypersecretion can not only alleviate clinical symptoms but also reduce the respiratory inflammatory response in COPD.^[5]^

Thus, research for a better understanding about molecular mechanisms of mucus hypersecretion has become a hotspot for the treatment of COPD.

## Risk factors of airway mucus hypersecretion

The most common exogenous factor that stimulates mucus hypersecretion is cigarette smoke.^[6]^ Previous studies have shown that cigarette smoke extract (CSE) can increase the expression of mucin-5 subtype AC(MUC5AC) in a concentration-dependent manner.^[7]^ Another common risk factor is particulate matter 2.5 (PM2.5), which can be deposited in the small airways and induce MUC5AC expression caused by oxidative stress.^[8,9]^

Most of endogenous factors that induce mucus hypersecretion are inflammatory mediators, including neutrophil elastase (NE),^[10]^ interleukin-13 (IL-13),^[11]^ interleukin- 1β (IL-1β),^[12]^interleukin-4 (IL-4),^[13]^ lipopolysaccharide(LPS),^[14]^ matrix metalloproteinase-9 (MMP-9),^[15]^ *etc*. To date, NE is the most potent secretagogue.^[16]^ NE can cleave pro-transforming growth factor (TGF)-α to release mature TGF-α, which can activate epidermal growth factor receptor (EGFR) signaling in a ligand-dependent manner. In addition, NE is locally released to induce goblet cell degranulation when neutrophils and goblet cells are in close contact.^[17]^

## Molecular mechanisms of airway mucus hypersecretion

### Signaling pathways leading to mucus overproduction

EGFR signaling pathway: EGFR is activated by exogenous reactive oxygen species (ROS) induced by cigarette smoke and endogenous ROS generated by neutrophils. The downstream of EGFR can activate the nuclear transcription factors like nuclear factor kB (NF-kB) to trigger the transcription of MUC gene.^[18]^ Moreover, EGFR can phosphorylate signal transducer and activator of transcription 6 (STAT6), resulting in the down-regulation of forkhead box A2 (FOXA2) expression, thereby promoting the proliferation of goblet cells and the secretion of mucus.^[19]^

NF-kB signaling pathway: Both *in vitro* and *in vivo*, tumor necrosis factor (TNF)-α-mediated mucin production is dependent on IКB kinase (IKK) and NF-kB. NF-kB can regulate a variety of genes, including the MUC gene. Sequence analysis of the MUC2 and MUC5AC promoter clones revealed the presence of NF-kB response elements.^[20]^ A study in mouse models of COPD has demonstrated that NF-kB is essential for mucus production.^[21]^

p38 mitogen-activated protein kinase (MAPK) signaling pathway: p38 kinase, a member of the MAPK family, is a highly conserved protein kinase that can be activated by many inflammatory signals. Hemophilus influenzae is a common pathogen of COPD, and studies have found that Hemophilus influenzae can upregulate MUC gene transcription through the activation of TLR2-MyD88-dependent p38 MAPK pathway.^[22]^

### Signaling pathways leading to mucus secretion and clearance dysfunction

Myristoylated alanine-rich C kinase substrate (MARCKS) protein signaling pathway: MARCKS is a key regulatory molecule that mediates the release of mucin granules from human bronchial epithelial (HBE) cells.^[23]^ Phosphorylation of MARCKS by protein kinase C(PKC) leads to its translocation to the cytoplasm, followed by dephosphorylation of MARCKS by protein kinase G (PKG). Dephosphorylated MARCKS can bind to the membrane of mucin granules, then MARCKS promotes the transport of mucin granules to the membrane for release.^[24]^

Soluble N-ethylmaleimide-sensitive factor attachment protein receptor (SNARE) protein signaling pathway: SNARE proteins can initiate vesicle fusion, regulate intracellular membrane fusion events, and participate in the fusion of mucin particles and cell membranes. Through interacting with Ca^2+^ sensor and other factors, SNARE proteins mediate Ca^2+^-triggered mucin exocytosis.^[25]^ Studies have shown that the most abundant SNARE protein in the airway epithelium is mainly vesicle-associated membrane protein 8 (VAMP8).^[26]^

Epithelial sodium channel (ENaC) signaling pathway: Airway surface hydration is mainly controlled by ENaC and cystic fibrosis transmembrane regulator (CFTR) pathways. Studies have shown that cigarette smoke-induced ROS can disrupt the function and activity of ENaC, thereby disrupting mucin hydration and reducing mucus expulsion.^[27]^

Ciliophagy signaling pathway: Normal ciliary structure and function help to transport the mucus from the airway to the larynx. A study has demonstrated that histone deacetylase 6 (HDAC6) and other regulators of the autophagic pathway can lead to cilia shortening and abnormal function, resulting in impaired mucociliary clearance during CS exposure.^[28]^

## Treatment of airway mucus hypersecretion

For the above-mentioned risk factors and signaling pathways of airway mucus hypersecretion, the current treatment principles include eliminating exogenous risk factors, inhibiting mucus synthesis and secretion, promoting mucus clearance and other physical expectoration therapy.^[29]^ It has also been summarized in the Table 1.

**Table 1:** Targets and relative inhibitors for the treatment of mucus hypersecretion in COPD

Target	Inhibitor(s)
Smoking, PM2.5	Smoking cessation, improvement of air quality
Mucus synthesis	
NE	NE inhibitors, batimastat, suramin
EGFR	EGFR tyrosine kinase inhibitors: AG1478, BIBX1522, ZD1839
	p38 MAPK inhibitors: SB-202190
p38 MAPK NF-kB	Macrolide antibiotics: erythromycin, flurythromycin
Mucus secretion	
MARCKS	MARCKS inhibitors: MARCKS-specific antisense oligonucleotides
SNARE	SNARE protein inhibitors: EGF-LHN-C
Mucus clearance	
Cholinergic pathway Mucus viscosity	Expectorants: Guaifenesin, hypertonic saline
P2Y2	Mucolytics: N-Acetyl Cysteine
	P2Y2 receptor agonists

COPE: Chronic obstructive pulmonary disease; NE: neutrophil elastase; EGFR: epidermal growth factor receptor; MAPK: mitogen-activated protein kinase; MARCKS: myristoylated alanine-rich C kinase substrate; SNARE: soluble N-ethylmaleimide-sensitive factor attachment protein receptor; NF-kB: nuclear factor kappa B.

### Elimination of exogenous risk factors

As described above, smoking and air pollution are important risk factors for COPD. Therefore, smoking cessation and improvement of air quality should be the primary measures to prevent and treat mucus hypersecretion in chronic airway inflammatory diseases.^[30]^

### Drugs that inhibit mucus synthesis

EGFR tyrosine kinase inhibitors: Cigarette smoke, PM2.5 and oxidative stress can all stimulate EGFR activation, resulting in increased mucin secretion. It can be seen that the EGFR signaling pathway plays a central regulatory role in mucus hypersecretion. Studies have shown that pretreatment with the selective EGFR tyrosine kinase inhibitor BIBX1522 can effectively prevent the secretion of MUC5AC in asthma models and in a rat model of TNF-α-induced mucus hypersecretion.^[31]^

Macrolide antibiotics: Studies have shown that macrolide antibiotics can significantly reduce LPS-induced expression of MUC5AC in rat bronchi and reduce neutrophil counts as well as mucin levels in bronchoalveolar lavage fluid, which may be caused by the inactivation of NF-kB.^[32]^ Another study has shown that macrolide antibiotics can quickly accumulate in neutrophils and macrophages, thus inhibiting the secretion of NE and mucus secretion.^[33]^

NE inhibitors: Studies have shown that the NE inhibitor ONO-5046 can reverse ozone-induced mucus hypersecretion and neutrophil aggregation in guinea pig models.^[34]^ The selective NE inhibitor ICI2000355 has also been shown to reverse mucus hypersecretion induced by ovalbumin sensitization in guinea pig models.^[35]^

p38 MAPK inhibitors: Administration of the p38 MAPK inhibitor (SB-202190) significantly improved IL-13-induced goblet cell hyperplasia *in vitro*.^[36]^

### Drugs that inhibit mucus secretion

MARCKS inhibitors: As previously mentioned, MARCKS mediates the intracellular movement and exocytosis of mucin particles.^[24]^ Studies have shown that MARCKS-specific antisense oligonucleotides can downregulate MARCKS levels in HBE cells and significantly attenuate PKC/PKG activation-induced mucin secretion.^[37]^

SNARE protein inhibitors: SNARE protein inhibitor (EGF-LHN-C) inhibits mucin secretion by inhibiting the fusion of mucin granules with the cell membrane.^[38]^

### Drugs that promote mucus clearance

Expectorants: Expectorants can stimulate the cholinergic pathway and increase the secretion of serous fluid from the bronchial submucosal glands to reduce mucus viscosity, resulting in promotion of mucus clearance.^[39]^

Mucolytics: With the characteristics of reducing mucus viscosity, mucolytics are the most widely used drugs for the treatment of mucus hypersecretion. For example, N-Acetyl Cysteine (NAC) can break disulphide bonds linking mucin polymers to reduce the viscosity of mucus.^[40]^

Purinergic receptor P2Y, G-protein coupled, 2 (P2Y2) receptor agonists: Studies have shown that the efficiency of P2Y2 receptor agonists to increase airway mucus clearance is significantly greater than that of hypertonic saline solution, which may be related to the increase of water secretion and ciliary beating frequency by P2Y2 receptor agonists.^[41]^

In addition to the above drug treatment, the symptoms of patients can be alleviated to a certain extent through autogenic drainage,^[42]^ high-frequency chest wall oscillation (HFCWO)^[43]^ and bronchoscopic treatment.^[44]^

## Summary

So far, some progress has been made in the molecular mechanisms and drug treatment of mucus hypersecretion in COPD. A variety of drugs have been found to have certain therapeutic effects on mucus hypersecretion. However, most of the above drugs are still in the experimental research stage and there is still a long way to go before they can be used in clinical treatment. Further investigation is urgently needed to develop understanding and therapies for airway mucus hypersecretion in COPD.
